# Effect of Escitalopram on White Blood Cells in Patients With Major Depression

**DOI:** 10.4021/jocmr2009.12.1275

**Published:** 2009-12-28

**Authors:** Fatih Canan, Ahmet Ataoglu

**Affiliations:** a Duzce University School of Medicine, Department of Psychiatry, Duzce, Turkey

## Abstract

**Background:**

Immunological dysfunctions in the course of depression are recently intensively investigated. Pharmacotherapy of depression is speculated to affect immune response. In this study, our objective was to investigate whether escitalopram treatment would affect white blood cells in patients with major depression.

**Methods:**

Fifteen patients (11 women and 4 men), meeting the criteria for a current episode of major depressive disorder, were participated. White blood cell (WBC), neutrophil (NEUT), lymphocyte (LYMPH), monocyte (MONO), eosinophyl (EO), and basophyl (BASO) levels were measured at the entry to the study. After 8 weeks of open-label treatment with the selective serotonin reuptake inhibitor escitalopram (10-20 mg/d), the patients were readmitted and the measurements were repeated.

**Results:**

At the end of the study, LYMPH was found to be significantly decreased compared to the baseline value after 8 weeks treatment with escitalopram (p < 0.001). There was not a significant change in WBC, NEUT, MONO, EO, and BASO parameters.

**Conclusions:**

The present study has shown that escitalopram increased LYMPH in patients with major depression according to these results, the possible treatment of depression with escitalopram must be carried out with caution, in patients with immunological disturbances.

**Keywords:**

Escitalopram; Major depression; White blood cells

## Introduction

Immunological dysfunctions in the course of depression are recently intensively investigated, but the results are inconsistent, possibly due to etiological variability of depression [1]. Both immunosuppression and pathological activation, similar to that observed in inflammation or autoimmune diseases, were reported [1,2]. It has been demonstrated that pharmacotherapy of depression results in normalization of some disturbed indices of immunoreactivity [2]. In this study, our objective was to investigate whether escitalopram treatment would affect white blood cells in patients with major depression.

## Patients and Methods

The study was conducted in an outpatient clinic from April 2008 through August 2008. Fifteen patients (11 women and 4 men), meeting the Diagnostic and Statistical Manual of Mental Disorders, 4th Edition (DSM-IV) criteria for a current episode of major depressive disorder (MDD) (age, mean ± SD, 37 ± 10 years) were recruited. The eligible patients were physically healthy, aged 18 years or older. All of them had a baseline Montgomery-Asberg Depression Rating Scale (MADRS) [3] total score of at least 22. White blood cell (WBC), neutrophil (NEUT), lymphocyte (LYMPH), monocyte (MONO), eosinophyl (EO), and basophyl (BASO) levels were measured at the entry to the study. Blood samples were drawn after a fasting period of 12 h. They were obtained on the morning before starting the first dose of escitalopram. After 8 weeks of open-label treatment with the selective serotonin reuptake inhibitor escitalopram (10-20 mg/d), the patients with depression were readmitted and the measurements were repeated. All the patients were newly diagnosed and were not treated for depression previously. Written, informed consent was obtained, and the Ethics Commitee of Duzce University, Medical School Local Ethical Commitee for Clinical and Laboratory Studies, Turkey approved the study protocol. The study was conducted in accordance with the Declaration of Helsinki. Statistical analysis was done by SSPS statistical software (SPSS for windows 15.0, Inc., Chicago, IL, USA). Data were tested for normal distribution using the KolmogorovSmirnov test. Paired t-test was used to determine whether there was a significant difference between basal and posttreatment variables. Data are expressed as mean ± standart deviation. Statistical significance was defined as p < 0.05.

## Results

At the end of the study, LYMPH was found to be significantly decreased compared to the baseline value after 8 weeks treatment with escitalopram (p < 0.001). There was not a significant change in WBC, NEUT, MONO, EO, and BASO parameters (Table 1).

**Table 1:** Baseline And Post Escitalopram Treatment Values of White Blood Cells

	Before treatment	After treatment	p value
WBC	6.93 ± 2.43	6.78 ±1.35	0.732
NEUT	4.46 ± 2.05	3.97 ± 0.94	0.218
LYMPH	1.82 ± 0.55	2.08 ± 0.49	0.008
MONO	0.57 ± 0.20	1.61 ± 0.68	0.167
EO	0.20 ± 0.41	0.30 ± 0.66	0.644
BASO	0.19 ± 0.01	0.20 ± 0.01	0.751

White Blood Cell (WBC), neutrophil (NEUT), lymphocyte (LYMPH), monocyte (MONO), eosinophyl (EO), and basophyl (BASO).

## Discussion

Numerous observations indicate that the changes in the state of the central nervous system, particularly of the mood level, are accompanied by the changes in immunological status of the organism, and conversely, the diseases involving immunological responses influence behavior [4]. Serotonin was found to modulate immunological responses such as proliferation of lymphocytes induced by mitogens [5,6]. Serotonin in lymphatic organs derives from macrophages, lymphocytes, basophils, mast cells and blood platelets, and also, secondarily, the noradrenergic sympathetic nerve endings, that are capable of serotonin reuptake [7]. Available data demonstrate that serotonin affects the immunological responses both in vitro and in vivo conditions, and its action is biphasic: low concentrations of serotonin stimulate, while its high levels suppress the immunological system. Thus, serotonin may be regarded as an autocrine and paracrine modulator of immunological responses [5,6].

The present study has shown that escitalopram increased LYMPH in patients with major depression. This study indicates that the escitalopram, a selective serotonon reuptike inhibitor antidepressant, seems to have a lymphocyte proliferative effect. According to these results, the possible treatment of depression with escitalopram must be carried out with caution, in patients with immunological disturbances.
